# Household Food Insecurity, Anemia, Malnutrition and Unfavorable Dietary Diversity among Adolescents: Quadruple Whammies in the Era of Escalating Crises in Lebanon

**DOI:** 10.3390/nu14245290

**Published:** 2022-12-12

**Authors:** Maha Hoteit, Hala Mohsen, Nour Yazbeck, Sarah Diab, Jessica Sarkis, Yonna Sacre, Lara Hanna-Wakim, Khlood Bookari

**Affiliations:** 1 Faculty of Public Health, Section 1, Lebanese University, Beirut 6573, Lebanon; 2 PHENOL Research Group (Public Health Nutrition Program Lebanon), Faculty of Public Health, Lebanese University, Beirut 6573, Lebanon; 3 Lebanese University Nutrition Surveillance Center (LUNSC), Lebanese Food Drugs and Chemical Administrations, Lebanese University, Beirut 6573, Lebanon; 4 University Medical Center, Lebanese University, Beirut 6573, Lebanon; 5 Department of Nutrition and Food Sciences, Faculty of Arts and Sciences, Holy Spirit University of Kaslik (USEK), Jounieh 1200, Lebanon; 6 Department of Agricultural and Food Engineering, School of Engineering, Holy Spirit University of Kaslik (USEK), Jounieh 1200, Lebanon; 7 Department of Clinical Nutrition, Faculty of Applied Medical Sciences, Taibah University, Madinah 42353, Saudi Arabia; 8 National Nutrition Committee, Saudi Food and Drug Authority, Riyadh 13513, Saudi Arabia

**Keywords:** adolescents, parents, dietary diversity, food insecurity, anemia, malnutrition

## Abstract

Adolescence is a transitional period between childhood and adulthood. Nowadays, adolescents in Lebanon are growing during a time of unprecedented health crises and political instability. This study aimed to determine the prevalence and correlates of malnutrition, dietary diversity in adolescents’ households, and adolescents’ self-reported food insecurity in Lebanon. A national representative sample of 450 parent–adolescent dyads (parents: mean age ± standard deviation (SD) = 46.0 ± 7.0, mothers: 59.0%; adolescents: mean age ± SD = 15.0 ± 3.0, girls: 54.6%) were interviewed. Anthropometric and blood hemoglobin measurements were performed for adolescents. The Food Consumption Score, the Arab Family Food Security Scale and the Adolescent-Level Food-Security Scale were used. The overall prevalence of adolescent stunting, thinness, overweight, obesity and anemia was 6.7%, 4.7%, 19.3%, 12.9% and 16.7%, respectively. Almost 40.4% and 68% of adolescent’s households consumed undiversified diets and were food insecure, respectively. Food insecurity (FI) affected 54.0% of adolescents. Adolescents attending schools (vs. university) were eight times more likely to be stunted (*p* = 0.04). Boys had a 4.3 times higher thinness risk (*p* = 0.005) compared to girls. Households reporting an income decline since the start of the Lebanese economic crisis were three times more likely to have a thin adolescent (*p* = 0.01). Parental overweight/obesity (*p* = 0.002) and lower education level (*p* = 0.04) nearly doubled the risk of adolescent overweight or obesity. At a time when escalating crises in Lebanon are shifting diets for the youngest generations, the development of adolescent-responsive nutritional policies becomes a must.

## 1. Introduction 

Adolescence, characterized by a period of rapid growth, is sensitive to malnutrition [1,2]. Simultaneously, many boys and girls start their adolescence stage undernourished, increasing their vulnerability to disease and early death [3]. Undernutrition throughout adolescence hinders physical growth, lowers academic performance and reduces economic productivity [4]. Meanwhile, overweight and obesity during this period of development are likely to remain into adulthood and promote chronic disease [4]. Furthermore, adolescents are most at risk for iron deficiency anemia (IDA) [3]. In 2019, IDA was the second greatest contributing cause of adolescent years lost to death and disability [3]. Despite these facts, addressing the nutrition requirements of adolescents has not been a prioritized topic in health and nutrition programming. Most nutrition initiatives targeted women and children under five [5]. Meanwhile, the adolescent generation’s nutrition status remains uninvestigated [6]. The World Health Organization (WHO) has advised that anthropometric measurements be performed on adolescents “at every opportunity,” [7]. Hence, anthropometric measurements, particularly body weight and height, are frequently used to assess adolescents’ nutrition status in both regular conditions and emergencies [8]. Moreover, under unstable conditions, it is important to consider other key factors such as eating habits and adolescent food security [8].

In Lebanon, adolescents are shouldering one of the world’s worst economic crises in recent years, propelling them toward risk of nutritional vulnerabilities and malnutrition. Lebanon is still recovering from its political instability, the economic constraints caused by the COVID-19 pandemic and the massive blast that hit the Beirut port on 4 August 2020 [9] and, most recently, the Ukraine–Russia war, all of which have heightened the risk of hunger and poverty for Lebanese people [10]. Lebanon has therefore developed into a typical nation where food insecurity (FI) exists and persists. Children and adolescents with FI have been observed as having higher school absenteeism and lower academic achievement [11]. Moreover, they negative emotions, concerns and empathy about their FI status [12]. Even though evidence shows that children and adolescents more accurately reflect FI than parent reports [12], to date, adolescents’ self-reported FI has not yet been evaluated in Lebanon. Additionally, no studies have explored the triple burden of malnutrition among Lebanese adolescents, with a current shortage of such data in the whole country. With this in mind, and to address the literature gaps, we conducted this study which includes a representative sample of Lebanese parent–adolescent dyads with the aim to: (1) determine the prevalence and correlates of malnutrition among age 10-19 Lebanese adolescents through anthropometric and blood hemoglobin measurements; (2) assess the dietary diversity (DD) of adolescents’ households; (3) document the prevalence of household-level FI and adolescents’ self-reported FI.

## 2. Materials and Methods

### 2.1. Study Design and Data Collection 

This cross-sectional study was conducted from March to July 2022. Study participants were recruited using the probability cluster sampling technique. As such, the clusters from where participants were recruited are the eight Lebanese governorates (Mount Lebanon, Beirut, South Lebanon, North Lebanon, Akkar, Beqaa, Baalbeck-Hermel and Nabatieh). Parent–adolescent dyads were recruited from each district using a probability proportional to size sampling technique. A single-population formula (*n* = [p (1 − p)] ∗ $[{\left(Z_{\propto /2}\right)}^{2}$/($e{)}^{2}$]) was used to determine the sample size, where *n* denotes the sample size, *Z* (∝/2) is the reliability coefficient of standard error at a 5% level of significance = 1.96, p represents the probability of youths being unable to practice preventive measures of the diseases (50%) and e refers to the level of standard error tolerated (5%) as stated by Hosmer and Lemeshow [13]. Based on this formula, it was determined that the minimum sample size of 400 respondents is sufficient to ensure appropriate power for statistical analyses. Subsequently, considering a non-response rate of around 10%, we reached a total of 450 adolescent–parent dyads. 

Our research team collaborated with numerous healthcare facilities, municipalities and pediatricians to schedule times and places to meet eligible parent–adolescent dyads for assessment. This study consisted of two stages: (i) stage 1 involved both parents and adolescents filling out an online self-administered questionnaire, and (ii) stage 2 involved meeting adolescents at the scheduled study locations to perform anthropometric and blood hemoglobin measurements.

### 2.2. Eligibility of Study Participants

For relevance to the study aims, we recruited participants as per the following eligibility criteria: (i) adolescents: aged 10–19 years old, healthy (having no chronic diseases), does not currently use iron or multivitamins dietary supplements, and did not donate blood in the month preceding data collection; (ii) parents: either parent, aged 18–64 years old; (iii) all participants had to be of Lebanese nationality. Moreover, we attempted to reach out to only one adolescent child per household.

### 2.3. Study Instrument

A well-structured questionnaire was used for collecting information from participants and was divided into multiple sections. The first section covered demographic and socioeconomic characteristics: (1) adolescents: date of birth, gender, residence, education level, working status, primary caregiver, previous use of iron dietary supplements (given that adolescents who were currently using iron supplements were not eligible to participate) and reason for iron dietary supplementation cessation; (2) parents: age, gender, self-reported body weight and height, education level, employment status, household’s monthly income, the impact of the Lebanese economic crisis on the household’s monthly income, number of co-residents per household and number of rooms (excluding the kitchen and bathrooms). Parents were categorized as being underweight, normal weight, overweight or obese based on their self-reported body weight and height and in accordance with the WHO standardized criteria [14]. 

One section of the questionnaire evaluated the household’s DD by calculating the food consumption score (FCS). Parent participants were asked to recall the foods they, or any family member, had consumed in the previous seven days before the survey. Each food item was given a score of 0 to 7 based on the number of days it was consumed. Afterward, food items were categorized into main food groups: starches and tubers, pulses, vegetables, fruits, meat products, oils and fats, dairy products and sugars. The formula to calculate the FCS based on these groups with the standard weights is: (starches × 2) + (pulses × 3) + vegetables + fruits + (meats × 4) + (dairy products × 4) + (fats × 0.5) + (sugar × 0.5). Households were then classified as having low DD (FCS ≤ 42) or high DD (FCS > 42) as per the World Food Program (WFP) suggested criteria [15]. 

Remaining parts of the questionnaire included the AFFSS [16], a score which was used to assess household food security. AFFSS was completed by parent participants and classified households into three severity levels of food security: food-secure, moderately food-insecure, and severely food-insecure. A valid 10-item scale [11] was introduced to adolescent participants allowing us to assess their food security. The scale classified adolescents into three levels in terms of their food security status: food-secure, moderately food-insecure, and severely food-insecure. In the current study, households or adolescents with “moderate” or “severe” FI were grouped under one “food insecure” category.

### 2.4. Anthropometric Measurements among Adolescent Participants 

A trained research team performed the anthropometric measurements for adolescents under standardized conditions. All measurements were repeated three times for each participant, and the average value was recorded. Body weight was measured to the nearest 0.1 kg using an electronic scale (AMBER Body scale, NUMED SARL, Beirut, Lebanon). Body weight measurements were performed with minimal clothing and barefooted. Height was measured to the nearest 0.1 cm using a portable stadiometer (Portable Height scale, NUMED SARL, Beirut, Lebanon). Waist and hip circumferences were measured using a measuring tape (BMIgirth measuring tape, NUMED SARL, Beirut, Lebanon). To measure the waist circumference, the tape was wrapped just above the hip bones of adolescents. For hip circumference measurement, the tape was placed at the widest part of the adolescent’s hip. Individualized waist-to-hip ration (WHR) was then obtained. The middle-upper arm circumference (MUAC) was measured to the nearest 0.1 cm, using a flexible non-stretch tape laid at the midpoint between the shoulder and the elbow. MUAC was transformed as a categorical variable using MUAC cut-off for 10–14 years and 15 years and above: 10–14 years old: normal (≥18.5 cm), moderate acute malnutrition (16.0–18.5 cm), and severe acute malnutrition (<16.0 cm); 15–19 years old: normal (≥22.0 cm), moderate acute malnutrition (18.5–22.0 cm), and severe acute malnutrition (<18.5 cm) [17]. Moreover, skinfold thickness (triceps, biceps, subscapular and suprailiac) was measured on the right side of the body to the nearest 0.1 mm using skinfold calipers (Accufat skinfold caliper, NUMED SARL, Beirut, Lebanon). 

The height-for-age Z score (HAZ) and body mass index (BMI)-for-age Z score (BAZ) were computed using the WHO Anthro-Plus software developed by the World Health Organization version 1.0.4. (Department of Nutrition, World Health Organization, Geneva, Switzerland). A cut-off value of <−2 standard deviation (SD) and <−3 SD for HAZ and BAZ classified adolescents as having moderate and severe stunting and thinness, respectively [8]. However, a cut-off value of >1 SD and >2 SD score for BAZ indicated overweight and obesity, respectively [8]. 

The obtained skinfold thickness values were used to calculate the % body fat (BF), according to an equation developed by Bray et al. (2001) [18]. The obtained % BF was used to calculate the total fat mass (TFM). Furthermore, based on the theory of the two-compartment body composition model, FFM was calculated by subtracting the TFM from total body weight.

(1) $$\begin{array}{c} \;BF\;\left(\%\right)\;\;\left(Boys\;and\;Girls\right)\\ \;\;\;\;\;\;\;\;\;\;\;=8.71+0.19\times \left(Subscapular\;\left(\mathrm{mm}\right)\right)\\ \;\;\;\;\;\;\;\;\;\;\;+0.76\times \left(Biceps\;\left(\mathrm{mm}\right)+0.18\times Suprailiac\;\left(\mathrm{mm}\right)\right)\\ \;\;\;\;\;\;\;\;\;\;\;+0.33\times \left(Triceps\;\left(\mathrm{mm}\right)\right) \end{array}$$

### 2.5. Anemia Assessment among Adolescent Participants

The Hgb blood level of adolescents was measured by a portable POC hemoglobin analyzer (CompoLab TS, Fresenius Kabi Deutschland GmbH, Bad Homburg, Germany). Per the WHO criteria [19], anemia was identified as a hemoglobin (Hgb) blood level of less than 11.5 g/dL in boys and girls younger than 12 years old, and an Hgb blood level of less than 12 g/dL in 12–14-year-old boys and girls. For those aged 15 years or older, a Hgb blood level of less than 13.0 g/dL indicated anemia among boys and an Hgb level less than 12.0 g/dL indicated anemia among girls. Adolescents were further categorized as having mild, moderate or severe anemia as the following: (1) mild (10–11 years old: Hgb level between 11 and 11.4 g/dL; 12–14 years old and girls aged ≥ 15 years old: Hgb level between 11 and 11.9; boys aged ≥15 years old: Hgb level between 11 and 12.9); (2) moderate (10–19 years old of both genders: Hgb between 8 and 10.9 g/dL); (3) severe (10–19 years old and both genders: Hgb level < 8 g/dL) [19]. 

### 2.6. Statistical Analysis 

Raw data, except the anthropometric data, were cleaned and exported to the Statistical Package of Social Sciences Software (SPSS) (Version 25.0, IBM Corp., Armonk, NY, USA) for analysis. Descriptive statistical measures, including frequencies, percentage, mean and standard deviation, were obtained to present and summarize the study findings. The Shapiro Wilk test was used to evaluate the normality of data. For non-normal distributed data, the non-parametric Mann–Whitney U test was used to detect mean differences for the variables with two groups. A bivariate analysis (χ^2^ test) was performed to document associations between study variables. In certain circumstances, when one or more of the cell counts in a 2 × 2 table were less than 5, the Fisher’s exact test was used instead. A multivariate analysis (the binary logistic regression) was performed to identify the principal correlates of adolescent nutrition status and adolescents’ self-reported FI. All tests were two-sided, and *p*-values of ≤0.05 were considered significant. 

## 3. Results 

### 3.1. Participant’s Sociodemographic Characteristics 

Of the total adolescent sample (*n* = 450), 54.7% were girls. The overall mean age ± SD (in years) of the adolescents was 15.0 ± 3.0. Additionally, 40% were young adolescents, 27.1% were middle-aged adolescents, and 32.9% were in the late adolescence stage. Similar to their parents, adolescents were representatively recruited from all Lebanese governorates as follows: Mount Lebanon (37.3%), Beirut (5.8%), South Lebanon (14.0%), North Lebanon (12.9%), Akkar (8.7%), Beqaa (6.9%), Baalbeck-Hermel (6.2%) and Nabatieh (8.2%). Most adolescents (79.6%) were school students. Almost all adolescents (93.6%) had both parents as primary caregivers. Only a few adolescents (6.9%) were working at the time of collecting data. Furthermore, 22.2% of adolescents had previously used iron dietary supplements. Adolescents stopped taking iron supplements for a variety of reasons, including: physician permission (37.0%), financial concerns (44.0%), loss of follow-up due to non-financial concerns (16.0%), and experiencing side-effects (3.0%) (Table 1).

As for the parents’ characteristics, of the 450 parent participants, 59% were mothers. The mean age ± SD (in years) of the overall parent sample was 46.0 ± 7.0. Further, 51.1% of the parents were middle-aged adults. Half of the parents (49.7%) were overweight or obese. Almost all parents were married (95.8%), and 54.0% had 2-3 children (54.0%). Just 7.8% of parents were uneducated, and 49.3% were unemployed. Additionally, 7.8% of the parents had no income, and 14.9% had an income of less than LBP 1.5 million. Moreover, 21.8% of parents reported that the Lebanese economic crisis caused a decline in their monthly income. Additionally, 45.0% of the sampled households were either crowded or overcrowded (household crowded index > 1) (Data not shown).

### 3.2. Anthropometric Characteristics, Body Composition Characteristics and Malnutrition Prevalence among Adolescents

The overall mean ± SD of adolescents’ body weight (kg), height (cm), WHR, HAZ, BAZ and MUAC (cm) were 53.1 ± 17.3, 156.3 ± 12.7, 0.79 ± 0.07, −0.3 ± 1.2, 0.3 ± 1.4 and 24.0 ± 4.4, respectively. Moreover, the overall mean ± SD of the triceps, biceps, subscapular and suprailiac skinfold thickness were 13.0 ± 6.0, 8.4 ± 4.6, 14.1 ± 8.3 and 13.7 ± 8.7, respectively. The overall mean ± SD of BF (%), TFM (kg) and fat-free mass (FFM) (kg) were 24.5 ± 7.8, 13.9 ± 8.7 and 39.2 ± 10.4, respectively. Moreover, an overall mean ± SD of 13.3 ± 1.6 for Hgb (g/dL) was reported among adolescents (Table 2).

Subsequently, our findings showed that 1.8% and 4.9% of the adolescents were severely and moderately stunted, respectively, indicating a 6.7% overall stunting prevalence. Furthermore, 0.7% and 4.0% of the adolescents had severe and moderate thinness, respectively, indicating an overall thinness prevalence of 4.7%. Overweight and obesity, on the other hand, were prevalent in 19.3% and 12.9% of adolescents, respectively. The MUAC value interpretation revealed that around 15% of adolescents had acute malnutrition, either moderate or severe. Nearly 17% of adolescents were anemic (moderate or severe: 33.4%) (Table 2). Among anemic adolescents, 44.0% reported previous use of iron dietary supplements; however, they stopped using the iron supplements due to financial concerns. Others reported doing so because of a loss of follow-up for non-financial concerns (40.0%) and due to the physician’s permission (16.0%) (data not shown). 

When stratified by gender, girls had significantly higher mean values of all skinfold thickness, BF (%) and TFM compared to boys. However, boys, in contrast to girls, had significantly higher mean values of height, FFM, and Hgb. Moreover, thinness was more prevalent in boys than girls (7.9% vs. 2.0%, *p* < 0.001) (Table 2).

### 3.3. The Frequency of Food Group Consumption and the Household’s DD Status

**Figure 1:**
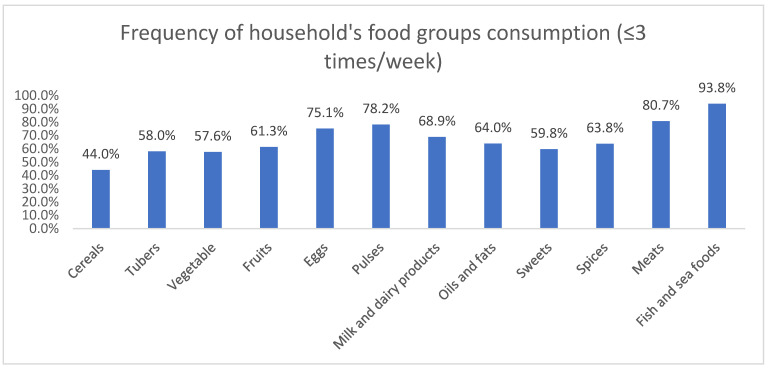
Frequency of foods group consumption (≤3 times/week) among participants’ households.

Parent participants were asked to recall the foods consumed by the family members over the previous 7 days before the survey to assess the households’ DD status. A significant proportion of households reported consuming vegetables (57.6%), fruits (61.3%), meat products (80.7%) and milk and dairy products (68.9%) across 3 days or fewer. This was also true for pulses (78.2%), oils and fats (64.0%) and sweets (59.8%), all of which were consumed across less than or equal to 3 days by most households (Figure 1). Subsequently, an overall mean FCS of 54.0 ± 29.0 was recorded for our study sample. However, 40.4% showed low FCS (<42), indicating an undiversified diet.

### 3.4. The Prevalence of Household FI and Adolescents’ Self-Reported FI

Our findings revealed that 68.0% and 54.0% of the households and adolescents were food insecure, respectively. The prevalence of household FI was the highest in Baalbeck-Hermel (82.2%), followed by Akkar (82%), Nabatieh (81.0%), Beqaa (80.7%), Beirut (69.2%), Mount Lebanon (67.9%), North Lebanon (62.0%) and South Lebanon (46.0%) districts. On the other hand, most food-insecure adolescents were from Akkar (84.6%), followed by North Lebanon (71.0%), Baalbeck-Hermel (64.3%), Beqaa (61.3%), Beirut (52.5%), Mount Lebanon (46.6%), Nabatieh (40.5%) and South Lebanon (36.6%) districts (data not shown). 

### 3.5. The Correlates Associated with the Adolescents’ Nutrition Status and Adolescents’ Self-Reported FI

In the current study, boys, in contrast to girls, had significantly higher thinness prevalence (7.8% vs. 2.0%, *p* = 0.004). Adolescents residing in Akkar had the highest prevalence of anemia (35.9%, *p* = 0.01). Adolescents with school-level education were more stunted (8.1%) than those who were university students (1.1%, *p* = 0.02). Parental weight status and education level significantly predicted adolescent child overweight/obesity, with 4 out of 10 overweight or obese parents (39.7%) also having an adolescent child with overweight or obesity, *p* = 0.002. Most adolescents with overweight or obesity were children of parents reporting school as the highest education level rather than a university (36.6 % vs. 26.8%, *p* = 0.01). Additionally, 9.2 % of households reporting an income decline since the start of the Lebanese economic crisis had thin adolescent children, *p* = 0.04. More than three quarters (84.6%) of adolescents in Akkar were observed to be food insecure, with the highest prevalence among all other governorates, *p* < 0.001. On average, 60% of adolescents living in crowded/overcrowded households were food insecure, *p* = 0.01. Furthermore, 63.8% of food-insecure households had adolescent children who self-reported FI, *p* = 0.001 (Supplementary Materials Table S1).

### 3.6. The Determinants of Adolescents’ Nutrition Status and Adolescents’ Self-Reported FI: Binary Logistic Regression Analysis 

Models 1, 2, 3, 4 and 5 present the significant correlates of adolescents’ stunting, thinness, overweight/obesity, anemia and self-reported adolescent FI, respectively. Adolescents with school-level education in contrast to university students were 8 times more likely to be stunted (odds ratio (OR) = 8.0, confidence interval (CI) = 1.1–59.6, *p* = 0.04). Moreover, male adolescents were 4.3 times more likely to have thinness (vs. girls; OR = 4.3, CI = 1.5–12.1, *p* = 0.005). Households reporting that the Lebanese economic crisis had caused a decline in their monthly income had a 3 times higher probability to have adolescent children with thinness (OR = 3.1, CI = 1.2–7.7, *p* = 0.01) (Table 3).

Overweight or obese parents had double the risk of having adolescent children with overweight or obesity (OR = 2.0, CI = 1.3–3.0, *p* = 0.002). Parents with a school education level (vs. university education level) were 1.4 times more likely to have overweight/obese adolescent children (OR = 1.4, CI = 0.9–2.3, *p* = 0.04). Adolescents residing in Akkar had more than 3 times higher risk of having anemia compared to those residing in other districts (OR = 3.2, OR = 1.6–6.5, *p* = 0.001). Moreover, adolescents residing in Akkar had a 4.4 times higher probability to be food insecure (OR = 4.4, CI = 1.8–11.1, *p* = 0.001). Adolescents living in crowded/overcrowded households were 1.3 times more likely to experience FI (OR = 1.3, CI = 0.9–2.0, *p* = 0.15). Lastly, household-level FI increased the risk of adolescent children experiencing FI by three-fold (OR = 3.4, CI = 2.2–5.2, *p* < 0.001) (Table 3).

## 4. Discussion 

The current study is the first of its kind on the nutrition status of Lebanese adolescents through anthropometric and blood hemoglobin measurements. Moreover, the DD of adolescents’ households, the households’ FI and the adolescents’ FI were also documented. The overall prevalence of adolescents’ stunting, thinness, overweight, obesity and anemia was 6.7%, 4.7%, 19.3%, 12.9% and 16.7%, respectively. Almost 40.4% of households consumed undiversified diets. FI affected 68% of households and 54.0% of adolescents. Adolescents attending schools (vs. university) were 8 times more likely to be stunted (*p* = 0.04). Male gender increased thinness risk by 4.3 times (*p* = 0.005). Households reporting an income decline since the start of the Lebanese economic crisis were 3 times more likely to have thin adolescent children (*p* = 0.01). Parental overweight/obesity (*p* = 0.002) and lower education level (*p* = 0.04) nearly doubled the risk of adolescent overweight or obesity. Adolescents residing in Akkar district had a 3 and 4 times higher risk of having anemia and FI, respectively (*p* = 0.001). Household FI tripled the likelihood of adolescents experiencing FI, *p* < 0.001.

The available literature shows that studies on adolescent undernutrition were, until recently, in short supply in almost all Arab countries, including Lebanon. However, our observed prevalence of stunting and thinness is lower than that reported in African and South Asian regions, including Bangladesh (49.0% and 40.0%, respectively) [20], India (8.4% and 23.7%, respectively) [21], Nepal (30.0% and 10.2%, respectively) [22] and Ethiopia (26.6% and 15.8%, respectively) [6]. On the other hand, it exceeded that observed among Russian (3.3% and 3.6%, respectively) [23] adolescents. In Lebanon, a recent national study showed that the prevalence of underweight, stunting and wasting among Lebanese children under age 5 was 0.5%, 8.4% and 6.7%, respectively [24]. These figures are likely to rise significantly as a result of Lebanon’s multiple conflicts, resulting in an increasing trend in adolescent undernutrition rates as poor nutritional status in childhood predisposes them to adolescent stunting. 

Additionally, the overall prevalence of adolescent overweight and obesity in the current study was 19.3% and 12.9%, respectively. At the national level, the current obesity prevalence is higher than that observed previously in Lebanon in 2011 (6.6%) [25]. Thus, obesity among Lebanese adolescents is experiencing an increasing trend over the years. In this context, Hwalla and colleagues [26] declared that the food consumption patterns of the Lebanese people revealed an increase in the intake of fat, milk products and animal protein. A Westernized diet has been frequently linked to obesity and metabolic diseases [26]. 

In the current study, 16.7% of adolescents had anemia. Preliminary national study findings showed a lower prevalence of iron-deficiency anemia among 8–18 year-old Lebanese adolescents (14.2%) [27]. Moreover, the current anemia prevalence has exceeded that reported in Kuwait (8.1%) [28], the United Arab Emirates (5.0%) [29] and Iraq (15.8%) [30]. However, it is much lower than that observed in India (71.7%) [31], Nepal (31.0%) [32] and Ethiopia (21.1%) [33]. The discrepancy in findings could be related to the variance in the sample population characteristics between adolescents recruited in each country, as some studies had included only female adolescents who usually have an additional risk of anemia after menarche occurrence [34]. Additionally, other risk factors such as the adolescence stage, poor diet and a family history of anemia could cause variances in anemia prevalence [35]. Amid the recurrent conflicts affecting Lebanon, people in the country consume inadequate diets lacking key micronutrients including iron and vitamin B12. Lebanon’s food prices have increased by 628% in just two years according to the World Food Program (WFP) [36]. Thus, this could limit the consumption of nutritious food and increase the risk of anemia, especially during adolescence when nutritional requirements are high. 

Additionally, the current study findings revealed that 40.4% of households were observed to be consuming undiversified diets. The proportion of households having poor DD in the current study remarkably exceeds that reported among the Lebanese population during the COVID-19 home isolation period, where 14.0% of households were observed as consuming an undiversified diet [37], but are consistent with that reported among Lebanese people in the period following the Beirut port explosion, where half of the Lebanese population had poor DD [38]. The latter findings are justified given that Lebanon is still recovering from its political instability, the economic meltdown caused by the COVID-19 pandemic, the massive blast that occurred in the Beirut port on 4 August 2020 and most recently the Ukraine war, all of which increased the risk of hunger and poverty for Lebanese citizens. 

Furthermore, our study showed that more than half of sampled households (68.0%) were food insecure. Starting with the impact of the Syrian conflict, the ensuing political crisis, the emergence of COVID-19 and the port explosion in Beirut, Lebanon has experienced a wave of numerous, unprecedented shocks. These events have all driven the Lebanese population into a state of hunger and FI [38]. The food crisis in Lebanon was made worse by the conflict in Ukraine, as 80% of the wheat in Lebanon is imported from Russia and Ukraine [39]. Moreover, in the current study, 54.0% of adolescents self-reported FI. This prevalence was higher than that observed among adolescents from 95 countries (30.0%) [40]. Hence, household-level FI increased the risk of adolescents’ FI in the current study. This finding is justified because FI, if present, can affect every member of the household, particularly the youngest. Thus, our findings urge the need to consider targeting Lebanese adolescents for nutrition-sensitive interventions, such as food assistance programs, to mitigate the detrimental consequences of FI. 

Additionally, our findings showed that adolescents attending schools (vs. university students) had higher susceptibility to stunted growth. This finding is in concordance with that reported in Ethiopia [41], where school-aged children were identified as a particular risk group for stunting. Additionally, male adolescents, in contrast to girls, were more likely to have thinness. This is consistent with recent review findings on the sex differences in undernutrition prevalence [42]. Households reporting an income decline since the start of the Lebanese economic crisis had greater probability of having thin adolescent children in the current study. This finding is warranted. Evidence shows that socioeconomic inequalities determine the prevalence of malnutrition in low- and middle-income countries [43]. Additionally, parental obesity represented the most potent risk factor for having obese children due to genetic and family environmental factors that trigger unhealthy eating habits [44]. Adolescents having parents with school-level education (vs. higher education) were at 1.4 times higher risk to be overweight/obese, *p* = 0.037. This finding might be strengthened by the latest research findings suggesting that the prevention of overweight and obesity among children and adolescents was more successful among highly educated families [45]. Furthermore, according to a recent systematic review, women’s empowerment has gained attention as critical for child and adolescent nutrition [46]. 

The inadequate focus on adolescent nutrition status impedes progress toward breaking the malnutrition intergenerational cycle. Context-specific nutrition-sensitive and nutrition-specific interventions for directing the household income, such as cash transfer, would be highly beneficial. School-based interventions including school feeding programs and focusing on adolescents’ nutrient requirements and diet preferences could also be considered. Even though multi-sectoral interventions and policy implementation are gaining traction worldwide, nutrition advocates in Lebanon are rarely active, resulting in missed opportunities to implement nutrition initiatives. Governments have a responsibility to establish functioning institutions and infrastructure that enable the poor to achieve nutrition security and to provide services for treatment and prevention of malnutrition and related diseases. Yet, despite high-level commitment in the context of the Millennium Development Goals (MDGs) and other initiatives, most developing countries are likely to fail in achieving their nutrition-related goals, although there are large differences in nutritional achievements across countries. 

### Study Limitations and Strengths

Findings from this study must be interpreted in light of some limitations. One limitation of the study is its design, which is cross-sectional, limiting our ability to draw causal pathways between study variables. Moreover, as the questionnaire used in data collection was self-administered, information bias could be assumed. Recall bias, as well, might mold the findings of the households’ DD and FI. Another limitation worth mentioning is the observer’s bias during the anthropometric measurements. On the other hand, the current study provides the first data on Lebanese adolescents’ nutrition status enrolling a nationally representative sample of parent–adolescent dyads. Thus, this study addresses research gaps and advocates for adolescents’ health. 

## 5. Conclusions

Adolescents in Lebanon bear the triple burden of malnutrition and the petrifying existence of FI. Stunting, thinness, overweight, obesity and anemia were all considerably prevalent among Lebanese adolescents. In addition to this, more than half the adolescents were food insecure and belong to households experiencing food insecurity, with many having poor dietary diversity. This calls for governments to prioritize policies and actions and allocate substantial investments in their efforts to address the needs of their malnourished populations.

## Figures and Tables

**Table 1:** Adolescents’ general characteristics.

	Overall (*n* = 450)		Girls (*n* = 246)		Boys (*n* = 204)		
	*n*	%	*n*	%	*n*	%	*p*-Value
Adolescence stage							<0.001 **
Young adolescents (10–13 years old)	180	40.0	86	35.0	94	46.1	
Middle-aged adolescents (14–16 years old)	122	27.1	55	22.4	67	32.8	
Older adolescents (17–19 years old)	148	32.9	105	42.6	43	21.1	
Residence							<0.001 **
Mount Lebanon	168	37.3	80	32.5	88	43.1	
Beirut	26	5.8	19	7.7	7	3.4	
South Lebanon	63	14.0	46	18.7	17	8.3	
North Lebanon	58	12.9	36	14.6	22	10.8	
Akkar	39	8.7	24	9.8	15	7.4	
Beqaa	31	6.9	18	7.3	13	6.4	
Baalbeck-Hermel	28	6.2	10	4.1	18	8.8	
Nabatieh	37	8.2	13	5.3	24	11.8	
Education							<0.001 **
School level	358	79.6	177	72.0	181	88.7	
University level	92	20.4	69	28.0	23	11.3	
Primary caregiver							0.04 *
Mother	10	2.2	7	2.8	3	1.5	
Father	7	1.6	4	1.6	3	1.5	
Both (mother and father)	421	93.6	224	91.1	197	96.6	
Other caregivers	12	2.7	11	4.5	1	0.5	
Currently working							0.27
No	419	93.1	232	94.3	187	91.7	
Yes	31	6.9	14	5.7	17	8.3	
Previous intake of iron dietary supplements							0.09
No	350	77.8	184	74.8	166	81.4	
Yes	100	22.2	62	25.2	38	18.6	
Reason for iron dietary supplements cessation (*n* = 100; among supplement users)							0.02 *
After physician permission/no longer needed	37	37.0	21	33.9	16	42.1	
Financial concerns	44	44.0	34	54.8	10	26.3	
Non-financial concerns (loss of follow-up with the physician)	16	16.0	6	9.7	10	26.3	
Experiencing side-effects	3	3.0	1	1.6	2	5.3	

* Significant at *p*-value < 0.05 for χ^2^ test; ** significant at *p*-value < 0.001 for χ^2^ test.

**Table 2:** Anthropometric characteristics, body composition characteristics and malnutrition prevalence among adolescent participants.

	Overall (*n* = 450)	Girls (*n* = 246)	Boys (*n* = 204)	*p*-Value
	Mean ± SD	Mean ± SD	Mean ± SD	
Body weight (kg)	53.1 ± 17.3	51.9 ± 14.6	54.7 ± 20.1	0.47
Height (cm)	156.3 ± 12.7	154.4 ± 10.0	158.5 ± 15.1	<0.001 *
WHR ^(1)^	0.79 ± 0.07	0.8 ± 0.1	0.8 ± 0.1	0.22
HAZ ^(2)^	−0.3 ± 1.2	−0.4 ± 1.1	−0.2 ± 1.2	0.10
BAZ ^(3)^	0.3 ± 1.4	0.3 ± 1.2	0.4 ± 1.6	0.57
MUAC (cm) ^(4)^	24.0 ± 4.4	23.9 ± 4.2	24.1 ± 4.6	0.55
Triceps skinfold thickness (mm)	13.0 ± 6.0	14.1 ± 5.3	11.6 ± 6.5	<0.001 *
Biceps skinfold thickness (mm)	8.4 ± 4.6	9.2 ± 4.4	7.5 ± 4.6	<0.001 *
Subscapular skinfold thickness (mm)	14.1 ± 8.3	14.8 ± 7.9	13.3 ± 8.7	0.002 *
Suprailiac skinfold thickness (mm)	13.7 ± 8.7	14.1 ± 8.1	13.2 ± 9.4	0.005 *
BF (%) ^(5)^	24.5 ± 7.8	25.7 ± 7.2	23.1 ± 8.3	<0.001 *
TFM (kg) ^(6)^	13.9 ± 8.7	14.1 ± 7.8	13.7 ± 9.7	0.03 *
FFM (kg) ^(7)^	39.2 ± 10.4	37.7 ± 8.1	41.0 ± 12.5	0.02 *
Hgb (g/dL) ^(8)^	13.3 ± 1.6	13.0 ± 1.3	13.6 ± 1.8	<0.001 *
	***n* (%)**	***n* (%)**	***n* (%)**	
HAZ classification				0.93
Normal (HAZ ≥ −2 SD to 3 SD)	416 (92.4)	226 (91.9)	190 (93.1)	
Severe stunting (HAZ < −3 SD)	8 (1.8)	5 (2.0)	3 (1.5)	
Moderate stunting (HAZ < −2 SD to −3 SD)	22 (4.9)	13 (5.3)	9 (4.4)	
Extreme tallness (HAZ > 3 SD)	4 (0.9)	2 (0.8)	2 (1.0)	
BAZ classification				<0.001 **
Normal (BAZ ≥ −2 SD to 1 SD)	284 (63.1)	170 (69.1)	114 (55.9)	
Severe thinness (BAZ < −3 SD)	3 (0.7)	0 (0)	3 (1.5)	
Moderate thinness (BAZ < −2 to −3 SD)	18 (4.0)	5 (2.0)	13 (6.4)	
Overweight (BAZ > 1 SD to 2 SD)	87 (19.3)	51 (20.7)	36 (17.6)	
Obese (BAZ > 2 SD)	58 (12.9)	20 (8.1)	38 (18.6)	
MUAC classification				0.75
Normal	382 (84.9)	209 (85.0)	173 (84.8)	
Moderate acute malnutrition	65 (14.4)	36 (14.6)	29 (14.2)	
Severe acute malnutrition	3 (0.7)	1 (0.4)	2 (1.0)	
Anemia Prevalence				0.80
Yes	75 (16.7)	40 (16.3)	35 (17.2)	
No	375 (83.3)	206 (83.7)	169 (82.8)	
Anemia severity (*n* = 75; among anemic)				0.27
Mild anemia	50 (66.6)	29 (72.5)	21 (60.0)	
Moderate anemia	24 (32.0)	10 (25.0)	14 (40.0)	
Severe anemia	1 (1.4)	1 (2.5)	0 (0.0)	

^(1)^ WHR: Waist-to-hip ratio; ^(2)^ HAZ: height-for-age Z score; ^(3)^ BAZ: BMI-for-age Z score; ^(4)^ MUAC: mid-upper arm circumference; ^(5)^ BF (%): body fat percentage; ^(6)^ TFM: total fat mass; ^(7)^ FFM: fat-free mass; ^(8)^ Hgb: blood hemoglobin level. * Significant at *p*-value < 0.05 for Mann–Whitney U test; ** significant at *p*-value < 0.05 for χ^2^ test. SD, standard deviation.

**Table 3:** The determinants of adolescents’ nutrition status and adolescents’ self-reported FI: binary logistic regression analysis.

Model 1: Binary Logistic Regression Taking the HAZ (No Stunting (Reference) vs. Stunting) as the Dependent Variable	OR (95% CI)	*p*-Value
Adolescents’ education level (Reference: university students)	1.0	-
School level	8.0 (1.1–59.6)	0.04
**Model 2: Binary Logistic Regression taking the BAZ (no thinness (reference) vs. thinness) as the dependent variable**		
Adolescents’ gender (Reference: girls)	1.0	-
Boys	4.3 (1.5–12.1)	0.005
The Lebanese economic crisis caused a decline in the household’s monthly salary (Reference: No)	-	-
Yes	3.1 (1.2–7.7)	0.01
**Model 3: Binary Logistic Regression taking the BAZ (no overweight/obesity (reference) vs. overweight/obesity) as the dependent variable**		
Parental weight status (Reference: Normal weight)	1.0	-
Overweight/obese	2.0 (1.3–3.0)	0.002
Parental education level (Reference: university level)	1.0	-
School level	1.4 (0.9–2.3)	0.04
**Model 4: Binary Logistic Regression taking the Anemia (No (reference) vs. Yes) as the dependent variable**		
Residence (Reference: Other governorates)	-	-
Akkar	3.2 (1.6–6.5)	0.001
**Model 5: Binary Logistic Regression taking the adolescents’ self-reported FI (food secure (reference) vs. food insecure) as the dependent variable**		
Residence (Reference: other governorates)	1.0	-
Akkar	4.4 (1.8–11.1)	0.001
Households’ crowding index (Reference: no crowding)	1.0	-
Crowded/overcrowded	1.3 (0.9–2.0)	0.15
Household-level FI (Reference: food-secure)	1.0	-
Food insecure	3.4 (2.2–5.2)	<0.001

FI, food insecurity; OR, odds ratio; CI, confidence interval.

## Data Availability

All the study data are reported in this paper.

## Supplementary Materials

Please use the standard format: The following are available online at https://www.mdpi.com/article/10.3390/nu14245290/s1, Table S1: The correlates associated with the adolescents’ nutrition status and adolescents’ self-reported FI.
