# Supplementary material for: Household Food Insecurity, Anemia, Malnutrition and Unfavorable Dietary Diversity among Adolescents: Quadruple Whammies in the Era of Escalating Crises in Lebanon
Source: Nutrients. 2022 Dec 12;14(24):5290. doi: 10.3390/nu14245290 (PMC9782497; doi:10.3390/nu14245290)
Supplement: Supplementary file 1 [file nutrients-14-05290-s001.zip › nutrients-2033111-SI.pdf]

Table S1. The correlates associated with the adolescents' nutrition status and adolescents' self-reported FI.

	**Stunting (HAZ < −2 SD)**		**Thinness (BAZ < −2 SD)**		**Overweight or Obesity (BAZ > 1 SD)**		**Anemia**		**Self-reported FI**						
	No	Yes	No	Yes	No	Yes	No	Yes	Food-secure	Food-insecure	*p*-value	*p*-value	*p*-value	*p*-value	*p*-value
	n (%)	*n* (%)	*n* (%)	*n* (%)	*n* (%)	*n* (%)	*n* (%)	*n* (%)	*n* (%)	*n* (%)	(a)	(b)	(c)	(d)	(e)
**Adolescents' gender**											0.54	**0.004**	0.09	0.80	0.59
Girl	228 (92.7)	18 (7.3)	241 (98.0)	5 (2.0)	175 (71.1)	71 (28.9)	206 (83.7)	40 (16.3)	116 (47.2)	13 (52.8)					
Boy	192 (94.1)	12 (5.9)	188 (92.2)	16 (7.8)	130 (63.7)	74 (36.3)	169 (82.8)	35 (17.2)	91 (44.6)	113 (55.4)					
**Adolescents' age**											0.13	0.48	0.70	0.85	0.65
Young adolescents	164 (91.1)	16 (8.9)	169 (93.9)	11 (6.1)	124 (68.9)	56 (31.1)	152 (84.4)	28 (15.6)	78 (43.3)	102 (56.7)					
Middle-aged adolescents	113 (92.6)	9 (7.4)	117 (95.9)	5 (4.1)	79 (64.8)	43 (35.2)	100 (82.0)	22 (18.0)	58 (47.5)	64 (52.5)					
Older adolescents	143 (96.6)	5 (3.4)	143 (96.6)	5 (3.4)	102 (68.9)	46 (31.1)	123 (83.1)	25 (16.9)	71 (48.0)	77 (52.0)					
**Residence**											0.58	0.73	0.12	**0.01**	**<0.001**
Mount Lebanon	153 (91.1)	15 (8.9)	158 (94.0)	10 (6.0)	108 (64.3)	60 (35.7)	144 (85.7)	24 (14.3)	90 (53.6)	78 (46.4)					
Beirut	26 (100.0)	0 (0.0)	25 (96.2)	1 (3.8)	20 (76.9)	6 (23.1)	22 (84.6)	4 (15.4)	10 (38.5)	16 (61.5)					
South Lebanon	60 (95.2)	3 (4.8)	60 (95.2)	3 (4.8)	47 (74.6)	16 (25.4)	57 (90.5)	6 (9.5)	40 (63.5)	23 (36.5)					
North Lebanon	56 (96.6)	2 (3.4)	57 (98.3)	1 (1.7)	40 (69.0)	18 (31.0)	43 (74.1)	15 (25.9)	17 (29.3)	41 (70.7)					
Akkar	36 (92.3)	3 (7.7)	37 (94.9)	2 (5.1)	23 (59.0)	16 (41.0)	25 (64.1)	14 (35.9)	6 (15.4)	33 (84.6)					
Beqaa	28 (90.3)	3 (9.7)	31 (100.0)	0 (0.0)	27 (87.1)	4 (12.9)	27 (87.1)	4 (12.9)	12 (38.7)	19 (61.3)					
Baalbeck-Hermel	27 (96.4)	1 (3.6)	27 (96.4)	1 (3.6)	18 (64.3)	10 (35.7)	25 (89.3)	3 (10.7)	10 (35.7)	18 (64.3)					
Nabatieh	34 (91.9)	3 (8.1)	34 (91.9)	3 (8.1)	22 (59.5)	15 (40.5)	32 (86.5)	5 (13.5)	22 (59.5)	15 (40.5)					
**Adolescents' education level**											**0.02**	0.27 *	0.09	0.92	0.87
School level	329 (91.9)	29 (8.1)	339 (94.7)	19 (5.3)	236 (65.9)	122 (34.1)	298 (83.2)	60 (16.8)	164 (45.8)	194 (54.2)					
University level	91 (98.9)	1 (1.1)	90 (97.8)	2 (2.2)	69 (75.0)	23 (25.0)	77 (83.7)	15 (16.3)	43 (46.7)	49 (53.3)					
**Primary caregiver**											0.55 *	0.78 *	0.30 *	0.38 *	0.40 *
Mother	10 (100.0)	0 (0.0)	10 (100.0)	0 (0.0)	6 (60.0)	4 (40.0)	9 (90.0)	1 (10.0)	2 (20.0)	8 (80.0)					
Father	7 (100.0)	0 (0.0)	7 (100.0)	0 (0.0)	4 (57.1)	3 (42.9)	6 (85.7)	1 (14.3)	3 (42.9)	4 (57.1)					
Both (mother and father)	391 (92.9)	30 (7.1)	400 (95.0)	21 (5.0)	284 (67.5)	137 (32.5)	348 (82.7)	73 (17.3)	197 (46.8)	224 (53.2)					
Other caregivers	12 (100.0)	0 (0.0)	12 (100.0)	0 (0.0)	11 (91.7)	1 (8.3)	12 (100.0)	0 (0.0)	5 (41.7)	7 (58.3)					
**Adolescents' employment**											0.25 *	0.65 *	0.42	0.11	0.08
No	393 (93.8)	26 (6.2)	400 (95.5)	19 (4.5)	286 (68.3)	133 (31.7)	346 (82.6)	73 (17.3)	188 (44.9)	231 (55.1)					
Yes	27 (87.1)	4 (12.9)	29 (93.5)	2 (6.5)	19 (61.3)	12 (38.7)	29 (93.5)	2 (6.5)	19 (61.3)	12 (38.7)					
**Parental gender**											0.92	0.52	0.88	0.86	0.20
Female	248 (93.2)	18 (6.8)	255 (95.9)	11 (4.1)	181 (68.0)	85 (32.0)	221 (83.1)	45 (16.9)	129 (48.5)	137 (51.5)					
Male	172 (93.5)	12 (6.5)	174 (94.6)	10 (5.4)	124 (67.4)	60 (32.6)	154 (83.7)	30 (16.3)	78 (42.4)	106 (57.6)					
**Parental age**											0.90	0.09	0.82	0.24	0.14
Middle aged adults	215 (93.5)	15 (6.5)	223 (97.0)	7 (3.0)	157 (68.3)	73 (31.7)	187 (81.3)	43 (18.7)	98 (42.6)	132 (57.4)					
Old adults	205 (93.2)	15 (6.8)	206 (93.6)	14 (6.4)	148 (67.3)	72 (32.7)	188 (85.5)	32 (14.5)	109 (49.5)	111 (50.5)					
**Parental weight status**											0.82*	0.31*	**0.002**	0.14	0.49*
Underweight	8 (100.0)	0 (0.0)	7 (87.5)	1 (12.5)	7 (87.5)	1 (12.5)	8 (100.0)	0 (0.0)	4 (50.0)	4 (50.0)					

Normal	204 (93.6)	14 (6.4)	210 (96.3)	8 (3.7)	163 (74.8)	55 (25.2)	175 (80.3)	43 (19.7)	94 (43.1)	124 (56.9)					
Overweight/ Obese	208 (92.9)	16 (7.1)	212 (94.6)	12 (5.4)	135 (60.3)	89 (39.7)	192 (85.7)	32 (14.3)	109 (48.7)	115 (51.3)					
**Parental marital status**											0.68 *	1.0 *	0.21 *	0.26 *	0.72 *
Married	402 (93.3)	29 (6.7)	410 (95.1)	21 (4.9)	293 (68.0)	138 (32.0)	357 (82.8)	74 (17.2)	199 (46.2)	232 (53.8)					
Divorced	8 (88.9)	1 (11.1)	9 (100.0)	0 (0.0)	4 (44.4)	5 (55.6)	8 (88.9)	1 (11.1)	3 (33.3)	6(66.7)					
Widowed	10 (100.0)	0 (0.0)	10 (100.0)	0 (0.0)	8 (80.0)	2 (20.0)	10 (100.0)	0 (0.0)	5 (50.0)	5 (50.0)					
**Number of children per household**											0.17	0.09	0.10	0.58	0.07
One child	34 (100.0)	0 (0.0)	34 (100.0)	0 (0.0)	19 (55.9)	15 (44.1)	30 (88.2)	4 (11.8)	13 (38.2)	21 (61.8)					
2–3 children	223 (91.8)	20 (8.2)	227 (93.4)	16 (6.6)	174 (71.6)	69 (28.4)	204 (84.0)	39 (16.0)	124 (51.0)	119 (49.0)					
More than 3 children	163 (94.2)	10 (5.8)	168 (97.1)	5 (2.9)	112 (64.7)	61 (35.3)	141 (81.5)	32 (18.5)	70 (40.5)	103 (59.5)					
**Parental education level**											0.83	0.50	**0.01**	0.66	0.28
Illiterate	33 (94.3)	2 (5.7)	34 (97.1)	1 (2.9)	30 (85.7)	5 (14.3)	31 (88.6)	4 (11.4)	12 (34.3)	23 (65.7)					
School level	271 (92.8)	21 (7.2)	280 (95.9)	12 (4.1)	185 (63.4)	107 (36.6)	243 (83.2)	49 (16.8)	134 (45.9)	158 (54.1)					
University level	116 (94.3)	7 (5.7)	115 (93.5)	8 (6.5)	90 (73.2)	33 (26.8)	101 (82.1)	22 (17.9)	61 (49.6)	62 (50.4)					
**Parental employment status**											0.65	0.87	0.76	0.21	0.98
Unemployed	206 (92.8)	16 (7.2)	212 (95.5)	10 (4.5)	152 (68.5)	70 (31.5)	180 (81.1)	42 (18.9)	102 (45.9)	120 (54.1)					
Employed	214 (93.9)	14 (6.1)	217 (95.2)	11 (4.8)	153 (67.1)	75 (32.9)	195 (85.5)	33 (14.5)	105 (46.1)	123 (53.9)					
**Household's monthly income**											0.75 *	0.23 *	0.22	0.28	0.09
None	33 (94.3)	2 (5.7)	35 (100.0)	0 (0.0)	21 (60.0)	14 (40.0)	26 (74.3)	9 (25.7)	12 (34.3)	23 (65.7)					
Less than 1.5 million L.B.P.	61 (91.0)	6 (9.0)	65 (97.0)	2 (3.0)	47 (70.1)	20 (29.9)	61 (91.0)	6 (9.0)	26 (38.8)	41 (61.2)					
≥1.5 million L.B.P.	274 (93.2)	20 (6.8)	276 (93.9)	18 (6.1)	207 (70.4)	87 (29.6)	243 (82.7)	51 (17.3)	143 (48.6)	151 (51.4)					
≤300 USD	34 (94.4)	2 (5.6)	36 (100.0)	0 (0.0)	20 (55.6)	16 (44.4)	30 (83.3)	6 (16.7)	14 (38.9)	22 (61.1)					
More than 300 USD	18 (100.0)	0 (0.0)	17 (94.4)	1 (5.6)	10 (55.6)	8 944.4)	15 (83.3)	3 (16.7)	12 (66.7)	6 (33.3)					
**Lebanese economic crises impact on household's income**											0.77	**0.04** *	0.52	0.76	0.34
No impact	305 (93.8)	20 (6.2)	313 (96.3)	12 (3.7)	218 (67.1)	107 (32.9)	269 (82.8)	56 (17.2)	152 (46.8)	173 (53.2)					
Decline	90 (91.8)	8 (8.2)	89 (90.8)	9 (9.2)	66 (67.3)	21 (32.7)	84 (85.7)	14 (14.3)	40 (40.8)	58 (59.2)					
Increase	25 (92.6)	2 (7.4)	27 (100.0)	0 (0.0)	21 (77.8)	6 (22.2)	22 (81.5)	5 (18.5)	15 (55.6)	12 (44.4)					
**Household crowding index**											0.56	0.85	0.85	0.87	**0.01**
No crowding	233 (94.0)	15 (6.0)	236 (95.2)	12 (4.8)	169 (68.1)	79 (31.9)	206 (83.1)	42 (16.9)	127 (51.2)	121 (48.8)					
Crowding/overcrowding	187 (92.6)	15 (7.4)	193 (95.5)	9 (4.5)	136 (67.3)	66 (32.7)	169 (83.7)	33 (16.3)	80 (39.6)	122 (60.4)					
**Household FI**											0.83	0.52	0.68	0.30	**<0.001**
Food-secure	134 (93.7)	9 (6.3)	135 (94.4)	8 (5.6)	95 (66.4)	48 (33.6)	123 (86.0)	20 (14.0)	96 (67.1)	47 (32.9)					
Food-insecure	286 (93.2)	21 (6.8)	294 (95.8)	13 (4.2)	210 (68.4)	97 (31.6)	252 (82.1)	55 (17.9)	111 (36.2)	196 (63.8)					
DD															
Households with diversified diet (FCS < 42)	171 (40.7)	11 (36.7)	174 (40.6)	8 (38.1)	117 (38.4)	65 (44.8)	146 (38.9)	36 (48)	85 (36.2)	97 (45.1)	0.663	0.822	0.191	0.144	0.053
Households with undiversified diet (FCS > 42)	249 (59.3)	19 (63.3)	255 (59.4)	13 (61.9)	188 (61.6)	80 (55.2)	229(61.1)	39 (52)	150 (63.8)	118 (54.9)					

(a) Significance level related to stunting; (b) Significance level related to thinness; (c) Significance level related to overweight/obesity; (d) Significance level related to anemia; (e) Significance level related to self-reported FI. Bold values are significant at *p*-value <0.05 for χ^2 or Fisher's exact test; * *p*-value determined based on Fisher's exact test (when one or more of the cell counts in a 2 × 2 table are less than 5. FI, food insecurity; DD, dietary diversity; FCS, food consumption score; HAZ, height-for-age Z score; BAZ, BMI-for-age Z score; SD, standard deviation.
